# Structural and functional characterization of buffalo oviduct-specific glycoprotein (OVGP1) expressed during estrous cycle

**DOI:** 10.1042/BSR20191501

**Published:** 2019-12-10

**Authors:** Suman Choudhary, Jagadeesh Janjanam, Sudarshan Kumar, Jai K. Kaushik, Ashok K. Mohanty

**Affiliations:** 1 Animal Biotechnology Centre, National Dairy Research Institute, Karnal 132001, Haryana, India; 2 Department of Developmental Neurobiology, St. Jude Children’s Research Hospital, Memphis, TN 38105, U.S.A.

**Keywords:** Chitinase-like proteins, Evolution, Gametes, Oviduct

## Abstract

Oviduct-specific glycoprotein (OVGP1) is a high molecular weight chitinase-like protein belonging to GH18 family. It is secreted by non-ciliated epithelial cells of oviduct during estrous cycle providing an essential milieu for fertilization and embryo development. The present study reports the characterization of buffalo OVGP1 through structural modeling, carbohydrate-binding properties and evolutionary analysis. Structural model displayed the typical fold of GH18 family members till the boundary of chitinase-like domain further consisting of a large (β/α)_8_ TIM barrel sub-domain and a small (α+β) sub-domain. Two critical catalytic residues were found substituted in the catalytic centre (Asp to Phe118, Glu to Leu120) compared with the active chitinase. The carbohydrate-binding groove in TIM barrel was lined with various conserved aromatic residues. Molecular docking with different sugars revealed the involvement of various residues in hydrogen-bonding and non-bonded contacts. Most of the substrate-binding residues were conserved except for a few replacements (Ser13, Lys48, Asp49, Pro50, Asp167, Glu199, Gln272 and Phe275) in comparison with other GH18 members. The residues Trp10, Trp79, Asn80, Gln272, Phe275 and Trp334 were involved in recognition of all six ligands. The α+β sub-domain participated in sugar-binding through Thr270, Gln272, Tyr242 and Phe275. The binding assays revealed significant sugar-binding with purified native and recombinant OVGP1. Phylogenetic analysis revealed that OVGP1 was closely related to AMCases followed by other CLPs and evolution of OVGP1 occurred through several gene duplications. This is the first study describing the structural characteristics of OVGP1 that will further help to understand its interaction with gametes to perform crucial reproductive functions.

## Introduction

Oviductal epithelial cells express specific macromolecular components that are secreted into the oviductal fluid (OF). The glycoproteins present in the OF creates a suitable milieu for sperm-oocyte interaction and developmental competence of zygotes. The oviduct-specific glycoprotein, also known as OVGP1, OGP or oviductin, is a major glycoprotein among the constituents of OF, which is synthesized and secreted by the non-ciliated cells of oviduct epithelium. It is a high molecular weight, oestrogen-dependent glycoprotein that is known to interact with gametes for final maturation and successful fertilization process [1].

OVGP1 belongs to the glycosyl hydrolase 18 (GH18) family that includes chitinases with chitin-hydrolyzing activity and chi-lectins possessing no enzymatic activity due to amino acid substitutions in the catalytic centre. However, these proteins have retained the property of carbohydrate-binding and hence have been named as chi-lectins [2,3]. GH18 family members are known to retain one of the most versatile folds in nature i.e. the TIM-barrel structure [3,4]. OVGP1 consists of two domains: the N-terminal chitinase-like domain and C-terminal mucin-like domain [5]. The chitinase-like domain of OVGP1 is mostly conserved across various species and shows significant amino acid similarity with chitinases like HCHT (Human chitotriosidase) and chitinase-like proteins such as HCgp-39, MGP-40 and other SPX-40 proteins [6–10]. The C-terminal mucin-like domain possesses contiguous Ser/Thr rich repeated units that is heavily O-glycosylated resembling the characteristic feature of mucins. Most of the variability in OVGP1 belonging to various species is observed due to variations in the length of C-terminal residues [5]. The sequencing of cDNA clone of OVGP1 in hamster revealed that this zona pellucida-associated glycoprotein is an intriguing chimeric molecule because it encloses regions of significant similarity with chitinase-related proteins as well as a carboxy terminal mucin-type domain [11].

The structure and organization of the OVGP1 gene have been described in mouse [12], hamster [13], humans [14] and rabbit [15]. The cDNA sequences of OVGP1 have been determined in different species including bovine [16], hamster [17], human [18], goat [19], mouse [20], sheep [21], pig [22], baboon [23], rhesus monkey [24] and buffalo [25]. The expression of OVGP1 mRNA and protein is regulated by ovarian steroids i.e. estrogen and luteinizing hormone (LH) that is dependent upon the stage of estrous cycle and early pregnancy [1]. This glycoprotein is specifically expressed within the oviduct during postovulatory phase of the estrous/menstrual cycle [26]. OVGP1 associates with the zona pellucida (ZP) and is present in the perivitelline space of oocytes and embryos during fertilization [27]. OVGP1 purified from the oviduct has positive effects on sperm capacitation, sperm-ovum binding, ovum penetration and embryo development [28]. In one of our previous studies, the native and recombinant OVGP1 in buffalo were reported to have a positive effect on various sperm characteristics and *in vitro* embryo development [25,29]. It was reported to enhance the *in vitro* fertilization rate and embryo development in goats [30]. When gametes were pre-treated with OVGP1, the fertilization rate in cattle increased significantly [1].

There are various reports that provide a detailed information on the functional role of OVGP1 in enhancing the reproductive efficiency in human and important livestock species including buffalo [1,29,30]. However, there is a lack of information on structural characteristics of OVGP1 at protein level. Despite of the availability of a detailed information on X-ray structures of various chi-lectins in different species, no reports are available on three-dimensional (3D) structure of OVGP1 or its individual domains yet. A detailed analysis of the 3D structural features of OVGP1 is essential to establish a structure–function relationship and for understanding the molecule in greater detail. In the present study, we report on (1) determination of 3D structural model of buffalo OVGP1 through molecular modeling; (2) elucidation of carbohydrate-binding properties of buffalo OVGP1 through computational molecular docking and experimental binding assays; and (3) evolutionary relationship of buffalo OVGP1 with other CLPs and chitinases of GH18 family.

## Materials and methods

### Homology modeling

The homology modeling and structure refinement of buffalo OVGP1 protein was done using methods and protocols described by Krieger et al. 2009 [31] by using the YASARA Dynamics and Structure 17.1.28 (Yet Another Scientific Artificial Reality Application). YASARA Structure consists of a complete homology modeling module that fully automatically takes all the steps from an amino acid sequence to a refined high-resolution model using a CASP8 approved protocol. The amino acid sequence of buffalo OVGP1 was retrieved from NCBI (National Center for Biotechnology Information) with accession no. AFN52414 [25]. The potential modeling templates were searched by running six PSI-BLAST iterations. The modeling speed was kept slow and the maximum *E*-value allowed during the PSI-BLAST run for template search was 0.5. The templates were ranked based on the alignment score and structural quality according to WHATCHECK [32]. Five alignment variations per template were allowed for finding the best template and alignment. If the alignment was certain, a single model was created for each template while in cases where alignment was ambiguous, alternative models were also created. The missing loops were built and optimized by allowing 50 conformations per loop. Disulfide bonds were modeled during the model building process in YASARA based on a disulfide bond energy function developed by Canutescu et al [33]. Hydrogen bonding network was also optimized and side-chain rotamers for all residues were fine-tuned considering the solvation effects, electrostatic interactions and knowledge-based packing interactions. An unrestrained refinement was run during the modeling procedure using the knowledge-based force fields ensuring that the refinement did not move the model in wrong direction. The steps were followed for all combinations of templates and alignments and quality indicators i.e. *Z*-scores were calculated for the resulting models. The *Z*-score has been defined as the weighted averages of the individual *Z*-scores (overall *Z*-scores = 0.145 × Dihedrals + 0.390 × Packing1D + 0.465 × Packing3D) that describes how far away is the quality of model in terms of standard deviations from the average high-resolution X-ray structure [31]. The more negative values indicate that the homology model is worse than a high-resolution X-ray structure and the quality *Z*-score value below −2.0 is considered bad. Finally, a hybrid model was built by iterative replacement of bad regions in the top scoring model by corresponding fragments from the other models.

The homology model was subject to further refinement using the md_refine.mcr macro of YASARA based upon the knowledge-based YASARA2 force field. The macro runs 500 ps simulation of the homology model and 20 snapshots were saved every 25 ps. All parameters were kept at the values defined by the macro. During the macro run, energy minimization of the model was carried out with combined steepest descent and simulated annealing by fixing the backbone atoms of the aligned residues to avoid potential damage to the model. This was followed by a full unrestrained all-atom simulated annealing minimization. The energy minimization first used implicit solvent during side-chains and loop optimization, while in the simulated annealing minimization, explicit solvent shell was used for fine tuning the model.

### Molecular dynamics (MD) simulations

The refined model was further subjected to molecular dynamics simulations at constant temperature (298 K) and pressure (1 bar) using the md_run.mcr macro of YASARA. The structure of the protein was simulated in an 8 × 6 × 5 nm rectangular box with periodic boundaries and filled with a water density of 0.997 g/ml. Berendsen barostat and thermostat were used to control the temperatures and pressures during simulations. The ion-concentration was kept at 0.9% NaCl. The MD was run for 50 ns with a time step of 2 fs. The trajectory coordinates were calculated using AMBER14 force-field and saved at every 100 ps. Three independent simulations were conducted by changing random seed numbers to assign different initial velocities to the system to ensure adequate conformational space sampling. The trajectories were analyzed using md_analyze.mcr macro and root mean square deviation (RMSD) values were calculated with respect to the starting model of buffalo OVGP1. The conformational changes were calculated as Root Mean Square Fluctuation (RMSF) values. The stereochemical quality checks of the model were carried out by using PROCHECK through PDBSum server [34]. Structural analysis and superimpositions were done using YASARA and PyMOL 1.3.

### Molecular docking

The docking protocol was set up and binding energy calculations were done by using molecular docking program AutoDock Vina [35]. The ligand structures used for docking were downloaded from Pubchem database. The docked conformations were ranked according to their binding energies (U total in kcal/mol). The docking energy values were calculated as the sum of the electrostatic, van der Waals energies and the flexibility of the ligand itself. Low docking energy indicates high binding ability. Analysis of the receptor–ligand interactions was done using Discovery studio visualizer, PyMOL 1.3 and Ligplot.

### Binding assays

#### Protein purification

Buffalo recombinant OVGP1 expressed in *Escherichia coli* and native OVGP1 from buffalo oviducts were purified to homogeneity as described in our previous paper [29]. Stock solutions of native and recombinant OVGP1 were prepared in phosphate buffer. Protein concentrations were estimated using Bradford assay [36].

#### Fluorescence spectroscopy measurements

The fluorescence quenching assay was performed according to the protocol described earlier [37]. Fluorescence spectra were recorded with a Varian Cary Eclipse fluorescence spectrofluorometer (Agilent Technologies, Singapore) equipped with an electro-thermal temperature controller. The intrinsic fluorescence emission spectra of native and recombinant OVGP1 were recorded from 290 to 500 nm upon excitation at 280 nm wavelength using a quartz cuvette of 1.0-cm path length. Excitation and emission slits were maintained at 5 nm and the scan speed was set to 100 nm/min. All spectra were recorded at a constant temperature of 298 K. Standard reaction mixtures were prepared using 10 μM solution of protein in 25 mM phosphate buffer saline, pH 7.2 to a final volume of 1 ml. Different sugar ligands i.e. GlcNAc (N-acetyl glucosamine), GalNAc (N-acetyl galactosamine), Man (mannose), (GlcNAc)_2_, (GlcNAc)_4_ and (GlcNAc)_4_ were prepared at different concentrations (5, 10 and 15 mM), pre-incubated with a fixed concentration (10 μM) of native and recombinant OVGP1 for about 1 h and spectra were obtained. Appropriate blanks corresponding to the buffer were subtracted to correct the background emission of fluorescence.

#### Chitin-binding assay

Chitin beads (New England Biolabs) were washed thrice and equilibrated in chitin binding buffer (50 mM Tris HCl, 300 mM NaCl, 1 mM EDTA, pH 7.5). The chitin beads were pelleted by centrifugation, mixed gently with purified native and recombinant OVGP1 protein solutions (∼100 μg/ml) and incubated for about 1 h in eppendorf tubes. The samples were centrifuged for 1–2 min at high speed and washed with chitin-binding buffer extensively to remove any unbound protein. Bound protein was eluted from the beads by boiling the samples in SDS–PAGE sample buffer for 5 min and centrifuged. The bound and unbound fractions were subsequently analyzed by SDS–PAGE detection and finally confirmed by Western blot. For Western blot, the membrane was incubated with goat polyclonal antibody named as oviductin (N-20) (Santa Cruz Biotechnology, INC.) at 1:500 dilutions. Oviductin (N-20) is an affinity purified goat polyclonal antibody raised against a peptide mapping at the N terminus of OVGP1 (also designated Mucin 9) of human origin. The secondary antibody was horseradish peroxidase-conjugated anti-goat IgG (Bangalore Genei) at 1:1000 dilutions. Immunoreactivity was detected by using the DAB system (Bangalore Genei, India).

### Phylogenetic analysis

To study the evolutionary relationship of OVGP1, the amino acid sequences of OVGP1 in different species and other GH18 family members were downloaded from NCBI GenBank. The sequences were aligned by Clustal W program using default parameters [38]. The phylogenetic tree was constructed by MEGA6 software [39] using Neighbor-joining method (NJ) based on *p*-distance substitution model [40]. The reliability of clustering patterns was tested by calculating bootstrap support values (1000 replications).

## Results

### Structural modeling

**Figure 1:**
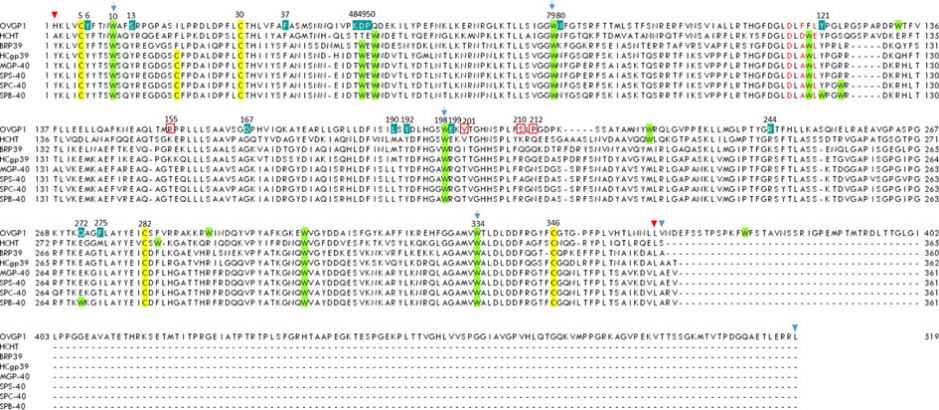
Multiple sequence alignment of buffalo OVGP1 with HCHT, BRP39, HCgp39, MGP-40, SPS-40, SPC-40, SPB-40 The start and end residues of chitinase-like domain and mucin-like domain of OVGP1 are indicated by inverted triangles in red and blue, respectively. The catalytic residues (Asp116, Phe118 and Leu120) are shown in red color. The conserved cysteine and tryptophan residues are highlighted with yellow and green color bars, respectively. The residues making up the wall of the barrel and attributed as being involved in sugar binding are indicated in blue. The conserved Trp10, Trp79, Trp198 and Trp334 involved in sugar-binding are indicated by arrows. The amino acid substitutions in comparison to other homologs are indicated by red boxes.

The structure of buffalo OVGP1 has not been solved in any of the species. It consists of a total of 540 amino acids where first 21 amino acids constitute the signal peptide. Buffalo OVGP1 consists of an N-terminal chitinase-like domain and a C-terminal mucin-like domain consisting of 361 and 158 amino acids, respectively (Figure 1). Homology model was built for 519 amino acids long matured form of buffalo OVGP1. For model building of full-length OVGP1, YASARA identified 10 possible templates after running 6 PSI-BLAST iterations (Supplementary Table S1). A total of 43 initial models were constructed from different alignment variants of identified templates and sorted by their overall quality *Z*-scores. The final hybrid model was built by iterative replacement of bad regions in the top scoring model by corresponding fragments from other models. The top scoring model was built from template 1HKK (high-resolution crystal structure of human chitinase in complex with allosamidin) and other models were built from templates 4TXG (Crystal Structure of a Family GH18 Chitinase from *Chromobacterium violaceum*) and 3RME (AMCase in complex with Compound 5) (Supplementary Table S2 and Figure S1). In the hybrid model, the region corresponding to chitinase-like domain was built from all the three templates i.e. 1HKK, 3RME and 4TXG, while the mucin-like domain was built from one template i.e. 4TXG. The region corresponding to mucin-like domain has not been reported yet in any of the mammalian GH18 family members except OVGP1. Therefore, the chitinase-like domain was modeled using templates from mammalian chitinases and mucin-like domain was modeled using template from a bacterial chitinase. A quality *Z*-score of −1.152 was obtained for the final hybrid model. The MD refinement of the hybrid model by YASARA2 force-field generated 20 snapshots and the best snapshot was selected with a quality *Z*-score of −0.49 and minimum energy of −27,4697.13 kcal.

The model structure was further subjected to all atom molecular dynamics (MD) simulations by optimizing its energy parameters and removing the structural strains. Large scale conformational changes were monitored through Root Mean Square Deviation (RMSD) measurements that showed the modeled structure to be in stable phase during three repeats of the MD simulation (Supplementary Figure S2A). The information about relatively rigid and flexible parts was attained by calculating the Root Mean Square Fluctuation (RMSF) per residue over a 50 ns trajectory (Supplementary Figure S2B). RMSF refers to the standard deviation of the atom position calculated from the average structure. The region covering the mucin-like domain (amino acids 401–512) was observed to be the most deviant. This region showed high RMSF values thus suggesting its less ordered or highly flexible structural configuration. The stereochemical quality check of the fully refined model showed that 87.9% residues were observed in the most favored regions, and 12.1% in the additionally allowed regions (Supplementary Figure S3). This indicates an acceptable overall geometry of the model where no residues were found in disallowed region of the Ramachandran plot. Further, the model showed good stereochemical property in terms of overall G-factor value of −0.14 calculated for dihedral angles and main chain covalent forces. Therefore, the overall model building and refinement process generated a final stable model of buffalo OVGP1 that was found to be suitable for further structural analysis.

### Overall structure of buffalo OVGP1

The overall structure of buffalo OVGP1 consists of 519 amino acids and comprises mainly two domains i.e. N-terminal chitinase-like domain (1–361 amino acids) and C-terminal mucin-like domain (362–519 amino acids).

#### Chitinase-like domain

**Figure 2:**
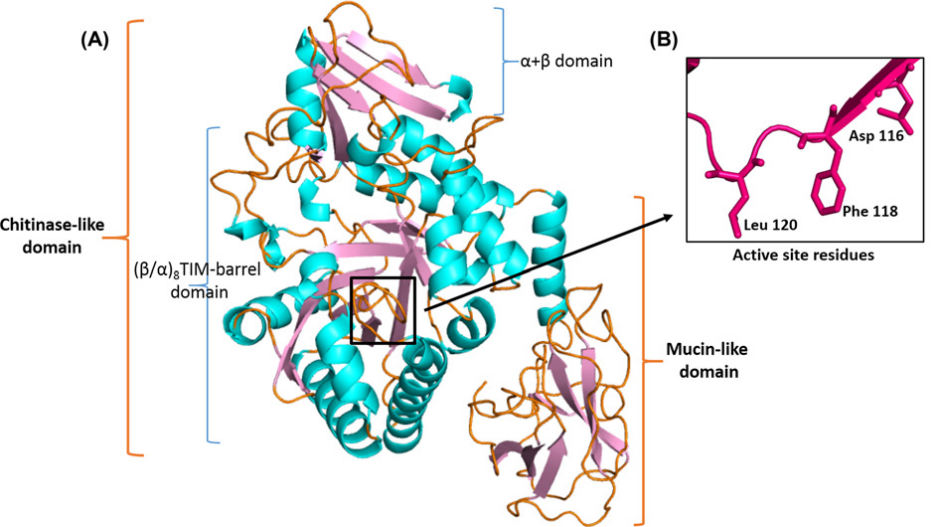
The overall structural display and catalytic centre of buffalo OVGP1 (**A**) The structure displays mainly two domains i.e. N-terminal chitinase-like domain and C- terminal mucin-like domain. The chitinase-like domain further consists of two sub-domains i.e. a large (β/α)_8_ TIM-barrel domain and a small α+β domain. α-helices are shown in cyan, β- strands in pink and connecting loops in orange color. The black squared box indicates the location of active site. (**B**) The catalytic triad of buffalo OVGP1 consisting of Asp116, Phe118 and Leu120.

The N-terminal chitinase-like domain region in the model represented the typical conserved fold as attained by members of GH18 family. It was further divided into two sub-domains i.e. a large (β/α)_8_ TIM barrel sub-domain and a small α+β sub-domain (Figure 2A). The (β/α)_8_ TIM barrel consisted of eight core parallel β-sheets surrounded by eight α-helices and possessed both N- and C- termini of chitinase-like domain. The TIM sub-domain comprised two polypeptide segments including residues 1–239 and 313–316. The polypeptide chain crosses over to form a small α+β sub-domain that includes residues 240–312 and consists of six antiparallel β-strands and one α-helix. Therefore, the TIM sub-domain included about 79% residues and small α+β sub-domain contained remaining 21% residues of chitinase-like domain of buffalo OVGP1. The eight β-strands of the barrel enclosed a tightly packed hydrophobic core that was formed entirely by the side chains of hydrophobic residues and eight α-helices were involved in forming the outer wall of this solenoid structure.

#### Mucin-like domain

The C-terminal mucin-like domain consisted of a total of 158 amino acid residues (362–519), thus, shorter in length than the N- terminal chitinase-like domain. The mucin-like domain was connected to the chitinase-like domain by 11 amino acids long loop structure. It comprised of one α-helix and seven antiparallel β-strands. The α-helix comprised 371–385 amino acid residues in continuation to the loop connecting the chitinase-like domain (Figure 2A). The six antiparallel β-strands were clustered into two groups connected by a long loop consisting of 30 amino acid residues (442–472). The structural topology of mucin-like domain was highly flexible with many loops.

### Active site architecture

**Figure 3:**
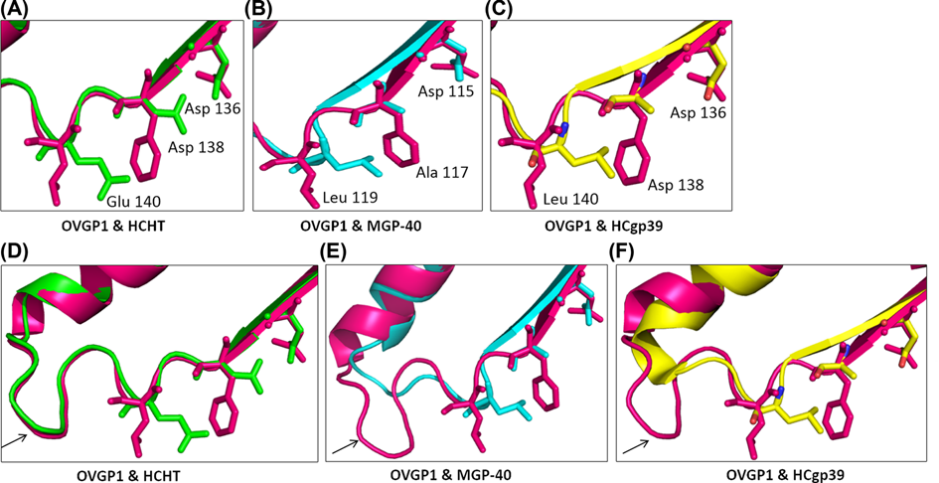
Comparison of the catalytic triad of buffalo OVGP1 with homologous structures (**A**–**C**) The catalytic triads of structural homologs i.e. HCHT (green), MGP-40 (cyan) and HCgp-39 (yellow), respectively in comparison with buffalo OVGP1. (**D**–**F**) The conformation of loop near the catalytic centre of buffalo OVGP1 in comparison with HCHT (green), MGP-40 (cyan) and HCgp-39 (yellow), respectively.

The catalytic centre of buffalo OVGP1 consists of Asp116, Phe118 and Leu120 (Figure 2B). The presence of an aromatic amino acid Phe118 within the catalytic center is a unique feature of OVGP1 that has not been observed in any of the other known crystal structures of chitinase-like proteins. The presence of Phe118 residue may impart more hydrophobicity within the catalytic center, thus influencing the ligand-binding interactions. The structural superpositions of the catalytic center of buffalo OVGP1 with other chitinase-like proteins of GH18 family represents various substitutions of catalytic residues (Figure 3A,C). In active human chitotriosidase (HCHT), the corresponding residues are Asp136, Asp138 and Glu140, respectively [41]. In chitinase-like proteins i.e. MGP-40, SPB-40 and all other SPX-40 structures [7–9], mutations were observed from Asp138 to Ala and Glu140 to Leu, respectively in comparison with active chitotriosidase resulting in loss of their chitinase activity. In other homologs like HCgp39 [41] and BRP39 [42], only one amino acid was found to be substituted i.e. Glu140 to Leu. A notable difference that was observed upon superimposition of buffalo OVGP1 structure with other CLPs and chitinases is a change in the conformation of a loop that is located near the active site. The loop conformation was found to be similar when compared with the active HCHT (Figure 3D). However, upon superimposition to CLPs like MGP-40 and HCgp39, a longer loop was observed with an entirely different conformation in buffalo OVGP1 (Figure 3E,F). The altered conformation of loop in OVGP1 can be attributed to a strong hydrogen bond interaction between the residues Leu124 and Ser127. In MGP-40, these residues have been replaced by Arg and Asp, respectively whereas in case of HCHT, both the residues have been replaced by Ser. The altered conformation of this loop in buffalo OVGP1 might have an influence on sugar-binding properties inside the binding pocket as compared with MGP-40.

### Carbohydrate binding properties of buffalo OVGP1

#### Molecular docking

**Figure 4:**
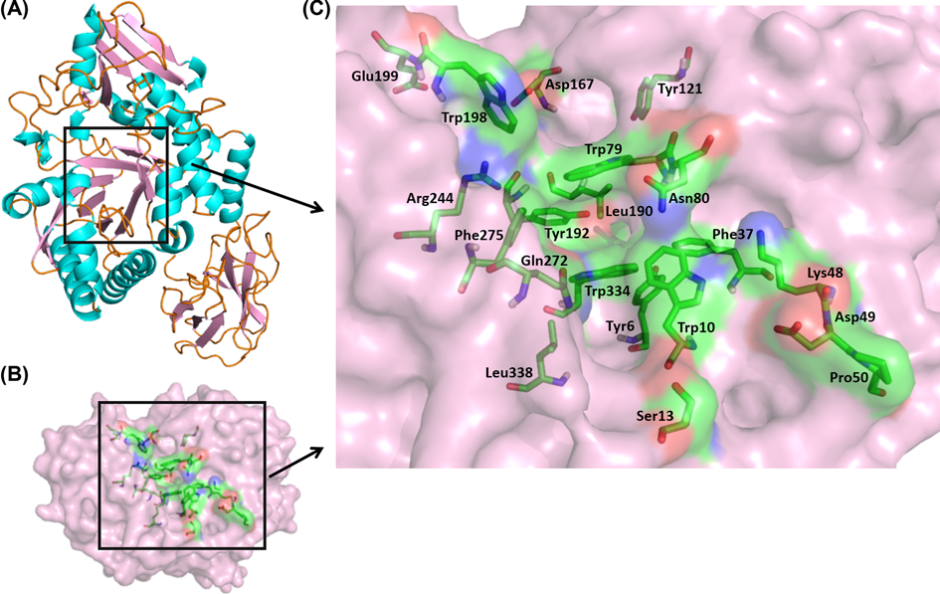
Substrate-binding pocket and its residues (**A**) Full-length structure of buffalo OVGP1 in cartoon representation showing the substrate-binding pocket located inside the TIM-barrel sub-domain as shown in square bracket. (**B**) Surface view of the substrate-binding pocket (green) shown inside the square bracket. (**C**) Residues in sticks representation located inside the substrate-binding pocket (green).

**Figure 5:**
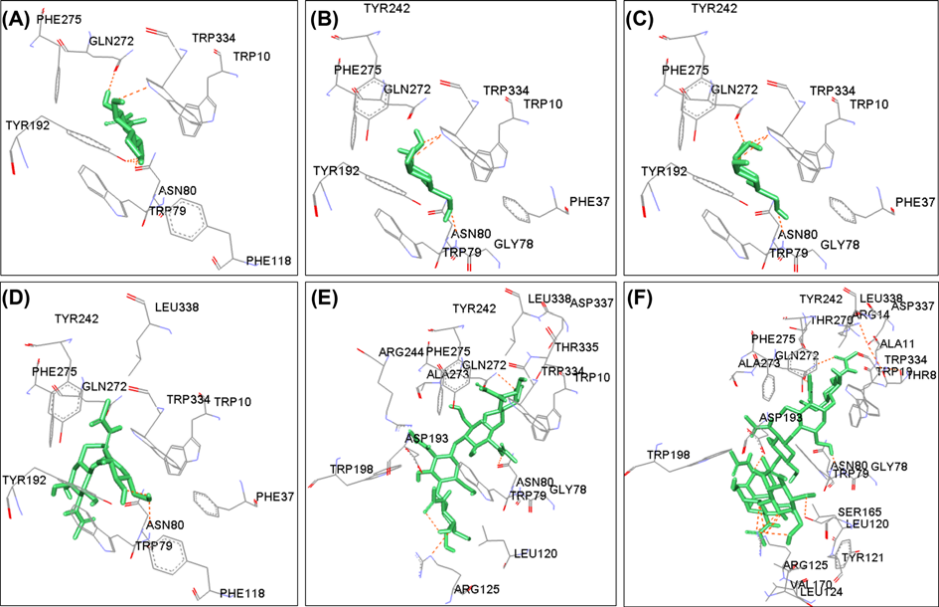
Interactions of buffalo OVGP1 with different sugar ligands A schematic representation of interactions of buffalo OVGP1 with (**A**) GlcNAc, (**B**) GalNAc, (**C**) Mannose, (**D**) (GlcNAc)_2_, (**E**) (GlcNAc)_4_ and (**F**) (GlcNAc)_6_. The ligands are shown in sticks representation (green). The residues involved in hydrogen-bonding and non-bonded interactions with side-chains of different ligands are shown in gray. Hydrogen bonds are indicated by dashed lines (orange) between the atoms involved.

The fully refined structure of buffalo OVGP1 was subjected to molecular docking with various saccharides/oligosaccharides, namely, GlcNAc, GalNAc, Man, (GlcNAc)_2_, (GlcNAc)_4_ and (GlcNAc)_6_. The docking analysis revealed the presence of a well formed carbohydrate-binding groove lined with various aromatic residues within the chitinase-like domain of buffalo OVGP1 (Figure 4). The docked structures reveal that the binding residues interact with different sugars through various hydrogen-bonded and non-bonded contacts (Figure 5). Aromatic residues are involved in various hydrophobic stacking interactions with the hydrophobic side chains of the bound sugar rings. The free energy (G) of binding and residues involved in hydrogen bonding and non-bonded contacts are listed in Table 1. It is noteworthy that in OVGP1-(GlcNAc)_6_ complex, the hexamer binds in a curved conformation as compared with the extended conformation of dimer and tetramer. This may be attributed to the presence of bulky residues with longer side chains within the groove resulting in less space to accommodate a six-residue long ligand in extended conformation. The residues Trp10, Phe37, Trp79, Asn80, Leu120, Tyr121, Tyr192, Asp193, Arg244, Gln272, Phe275, Trp334 and Leu338 interacted with ligands through various polar and non-polar contacts. It was notable that Trp10, Trp79, Asn80, Gln272, Phe275 and Trp334 participated in recognition of all six ligands. These residues were conserved in buffalo OVGP1 except Gln272 and Phe275 where Glu and Ile residues were found at these positions in SPS-40. Some of the residues such as Thr270, Gln272, Tyr242 and Phe275 located in the α+β sub-domain also take part in sugar-binding through both polar and non-polar contacts. Trp10 makes hydrophobic stacking interactions with all the six ligands i.e. GlcNAc, GalNAc, Man, (GlcNAc)_2_, (GlcNAc)_4_ and (GlcNAc)_6_. Another important residue Trp79 is found conserved across family 18 members in substrate-binding pocket of chitinases and CLPs including OVGP1. This residue has been considered crucial for sugar binding [6]. The hydroxyl group of Trp79 made hydrogen bonds with oxygen atoms of three ligands i.e. GalNAc, Man and (GlcNAc)_2_ whereas it interacted through hydrophobic interactions with GlcNAc, (GlcNAc)_4_ and (GlcNAc)_6_ ligands. Asn80 interacted with all ligands by formation of strong hydrogen bonds. It contributed two hydrogen bonds to OVGP1 complexes with GalNAc and Man. Arg125 made hydrogen bonds with (GlcNAc)_4_ and (GlcNAc)_6_ ligands. It was involved in an extensive interaction with (GlcNAc)_6_ by forming upto six hydrogen bonds to stabilize the OVGP1–(GlcNAc)_6_ complex. Another conserved residue Tyr192 makes a hydrogen bond through its hydroxyl group with oxygen atom of GlcNAc molecule, whereas it was involved in hydrophobic interactions with all other ligands such as GalNAc, mannose, (GlcNAc)_2_, (GlcNAc)_4_ and (GlcNAc)_6._ The NE1 atom of Trp334 made a single hydrogen bond with O atom of GlcNAc molecule whereas it contributed upto two hydrogen bonds with oxygen atom of (GlcNAc)_2_ and three hydrogen bonds with oxygen atoms of GalNAc and mannose ligands. Trp334 interacted with (GlcNAc)_4_ and (GlcNAc)_6_ ligands through hydrophobic interactions. Ser165 and Asp193 were involved in the formation of two hydrogen bonds with the GlcNAc moieties. The residue Trp198 interacted with (GlcNAc)_4_ and (GlcNAc)_6_ ligands through hydrophobic interactions. We observed that position 272 is occupied by Gln residue in OVGP1 in place of negatively charge Glu residue found in MGP-40 and HCgp39 chitinase-like proteins. This Gln272 interacted with all six ligands through one or two hydrogen bonds. It could make upto four hydrogen bonds with (GlcNAc)_4_ to stabilize the complex structure.

**Table 1:** Binding energy and residues involved in hydrogen bonding and non-bonded contacts in complex structures of buffalo OVGP1 with sugar ligands

Ligand	Binding energy (Kcal/mol)	Residues involved in hydrogen-bonded contacts	Residues involved in non-bonded contacts
GlcNAc	−4.25	Trp 79, Asn 80, Tyr 192, Gln 272, Trp 334	Trp 10, Trp 79, Phe 118, Phe 275
GalNAc	−4.07	Trp 79, Asn 80, Tyr 192, Gln 272, Trp 334	Trp 10, Phe 37, Asn 80, Tyr 192, Tyr 242, Phe 275, Trp 334, Leu 338
Mannose	−4.0	Trp 79, Asn 80, Gln 272, Trp 334	Trp 10, Phe 37, Asn 80, Tyr 192, Tyr 242, Phe 275, Trp 334
GlcNAc_2_	−5.01	Trp 79, Tyr 192, Tyr 242, Gln 272, Trp 334	Trp 10, Phe 37, Asn 80, Phe 118, Tyr 192, Phe 275, Tyr 242, Leu 338
GlcNAc_4_	−4.97	Asn 80, Arg 125, Trp 198, Gln 272, Ala 273, Thr 335	Trp 10, Gly 78, Asn 80, Trp 79, Leu 120, Arg 125, Asp 193, Tyr 242, Arg 244, Gln 272, Phe 275, Trp 334, Thr 335, Asp 337, Leu 338
GlcNAc_6_	−1.6	Thr 8, Arg 14, Trp 79, Asn 80, Arg 125, Ser 165, Asp 193, Trp 198, Gln 272, Trp 334	Trp 10, Ala 11, Arg 14, Gly 78, Tyr 121, Arg 125, Ser 165, Val 170, Trp 198, Leu 120, Tyr 121, Leu 124, Tyr 242, Thr 270, Gln 272, Phe 275, Asp 337, Leu 338

#### Fluorescence quenching assays

**Figure 6:**
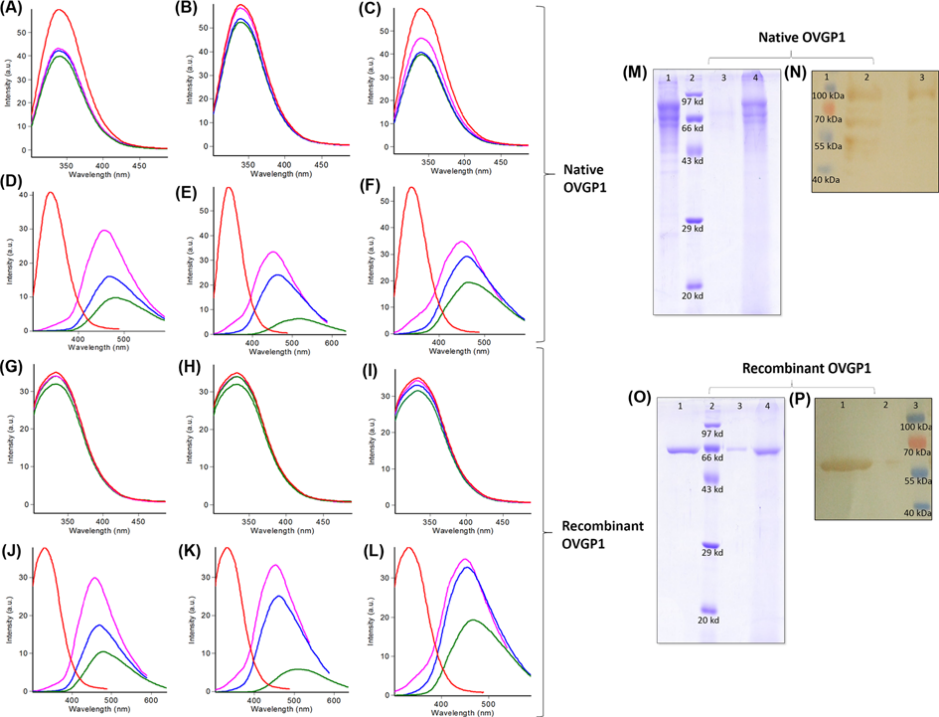
Experimental binding analysis of buffalo OVGP1 with different sugar ligands Fluorescence emission spectra of (**A**–**F**) native OVGP1 and (**G**–**L**) recombinant OVGP1 upon incubation with different concentrations of GlcNAc, GalNAc, Man, (GlcNAc)_2_, (GlcNAc)_4_ and (GlcNAc)_6_. Spectra for native and recombinant OVGP1 are shown in red. Spectra obtained at different concentrations of ligands i.e. 5, 10 and 15 mM are shown in pink, blue and green, respectively. (**M**) SDS–PAGE showing chitin-binding property of native OVGP1: lane 1 represents the purified proteins, lane 2 represents the protein molecular weight standard, lane 3 represents the bound fractions to chitin beads and lane 4 represents the unbound fractions after washing. (**N**) Western blot confirmation of chitin binding by native OVGP1 (lane 1: protein molecular weight standard, lane 2: bound fraction and lane 3: unbound fraction). (**O**) SDS–PAGE showing chitin-binding property of recombinant OVGP1: lane 1 represents the purified proteins, lane 2 represents the protein molecular weight standard, lane 3 represents the bound fractions to chitin beads and lane 4 represents the unbound fractions after washing. (**P**) Western blot confirmation of chitin binding by recombinant OVGP1 (lane 1: bound fraction, lane 2: unbound fraction and lane 3: protein molecular weight standard).

The analysis of sugar binding groove of OVGP1 reveals that four Trp residues at positions 10, 79, 198 and 334 are located within the binding site and involved in binding interactions (Supplementary Figure S4). Therefore, the binding ability of native and recombinant OVGP1 with GlcNAc, GalNAc, mannose, (GlcNAc)_2_, (GlcNAc)_4_ and (GlcNAc)_6_ ligands was analyzed by following changes in the fluorescence emission spectra of both the proteins in absence and presence of the sugars. A fixed concentration of protein sample was used with varying concentrations of ligands (5, 10 and 15 mM). As observed from the data (Figure 6A,L), a concentration-dependent binding was observed for different sugars. The fluorescence intensity of both native and recombinant OVGP1 decreased with increasing concentrations of ligands and a higher quenching was observed at 15 mM concentration in case of all ligands. In case of monosaccharides i.e. GlcNAc, GalNAc and mannose complexes of both native and recombinant OVGP1, the fluorescence intensity decreased without any shift in the emission maxima or change in the peak shape. However, for dimer (GlcNAc)_2_, tetramer (GlcNAc)_4_ and hexamer (GlcNAc)_6_ complexes of native and recombinant OVGP1, we observed a shift in the emission maxima towards longer wavelength i.e. red shift or bathochromic shift along with a decrease in the fluorescence intensity. This measurable change in the fluorescence spectrum might be due to a change in the solvent polarity, i.e. reorientation of the solvent molecules around the fluorophore when it is excited or due to the possible conformational changes within the binding site affecting the environment around the flourophores [43].

#### Chitin-binding assay

To further confirm the presence of a functional chitin-binding domain in both native and recombinant buffalo OVGP1, we carried out the chitin-binding assay. In SDS–PAGE and Western blot analysis, distinct protein bands in bound fractions for both native (∼55–90 kDa) and recombinant OVGP1 (∼58 kDa) were observed at their expected molecular weights (Figure 6M,P). In unbound fractions, faint bands were observed; however, the proteins appeared to interact with chitin beads efficiently as the protein was not removed even after extensive washing. This reveals the presence of a functional chitin-binding domain in both native and recombinant OVGP1.

### Comparison with chitinases and other chitinase-like proteins of GH18 family

The 3D structure of buffalo OVGP1 was superimposed over other members of GH18 family i.e. HCHT [6], MGP-40 [7], SPS-40 [9], SPC-40 [8] and HCgp39 [41] showing a sequence identity of 51%, 48%, 48%, 47% and 47%, respectively. The superimpositions delivered RMSD values of 0.579 A° (307 Cα atoms), 0.757 A° (299 Cα atoms) and 0.764 A° (312 Cα atoms), respectively. Upon superposition, a few structural differences were observed in chitinase-like domain in comparison with other structural homologs. These include various amino acid substitutions in the active site or substrate-binding pocket, changes in some loop conformations, changes in secondary structural features like smaller size of strands and helices and some extra strands of loops.

A comparison of carbohydrate-binding groove of buffalo OVGP1 with other CLPs like HCgp39 [41], MGP-40 [7], SPB-40 [10] and other SPX-40 structures [8,9] and chitinases [41] revealed some highly conserved residues such as Trp10, Trp79, Asn80, Arg125, Tyr192 and Trp334. However, some selective variations were observed that seemed to have altered the shape, size, charge and binding capabilities of sugar-binding groove. The residues Tyr34, Glu70, Trp71, Gly181, Phe208, Arg213 (HCgp39 numbering) were found well conserved in HCgp39, MGP-40,SPC-40 and HCHT structures. In buffalo OVGP1, these residues were found substituted with Ser13, Asp49, Pro50, Asp167, Leu194 and Glu199, respectively. Trp49 (Hcgp39 numbering) is conserved in HCgp39, MGP-40 and other SPX-40 structures except HCHT and buffalo OVGP1 where it is replaced by Thr49 and Asp49, respectively. The residues Tyr34, Trp69, Trp71, Gly181 and Phe208 in HCgp39 structure are involved in hydrophobic interactions with the hexasaccharide while Glu70 and Arg213 with strong polar contacts [41]. When compared with carbohydrate interacting residues of SPS-40, several amino acid substitutions were observed in buffalo OVGP1 including Tyr13 to Ser13, Trp48 to Lys48, Glu49 to Asp49, Trp50 to Pro50, Glu269 to Gln272 and Ile272 to Phe275. The residues Gln272 and Phe275 forms a part of smaller α+β domain in OVGP1.

Another notable difference in buffalo OVGP1 structure in comparison with other chitolectins and chitinases is the replacement of a free Cys20 residue inside the hydrophobic pocket by Ile20 residue. In buffalo OVGP1, four cysteine residues are present in comparison with five cysteine residues present in its homologs and involved in the formation of two disulfide bridges i.e. Cys5-Cys30 and Cys282-Cys346. The latter disulfide bond is positioned between two domains that serve to hold the two domains together. In other CLPs such as HCgp39 [41], MGP-40 [7] and other SPX-40 structures [8,9], a free Cys20 is present that is not involved in any disulfide bond formation and found buried in a tightly packed hydrophobic pocket.

The OVGP1 homolog, HCgp39, is known to exist either as dimer or tetramer and a critical interaction between Lys148 and Thr108 has been considered responsible for the dimeric or tetrameric state of this protein [41,44]. Buffalo OVGP1 and bovine SPC-40 exist as monomers [8,29], where Lys148 is replaced by Arg155 and Thr148, respectively. Thr, at position 108 in HCgp39, is found conserved in all the three homologous structures. The SPX-40 proteins i.e. SPS-40 (sheep) and SPC-40 (bovine) contain three overlapping flexible loops i.e. His188–His197 (loop1), Phe202–Arg212 (loop2) and Phe244–Pro260 (loop3) [8,9]. These loops at corresponding positions in HCgp39 showed strong intermolecular interactions and further contribute to its dimerization state [44]. We observed various amino acid substitutions in buffalo OVGP1 at corresponding positions. The residue His188 forms a strong hydrogen bond with Thr194 within the flexible loop2 of HCgp39. However, no such interaction was observed in OVGP1 as the corresponding His residue attained a different conformation, while Thr194 is substituted by non-polar Val at position 201. The hydrogen bond was also found absent in SPC-40 due to different conformations of the corresponding residues [8]. The polar Asn205 residue that is hydrogen bonded to Asp292 in SPC-40 is substituted by non-polar Proline at position 212 in buffalo OVGP1. The interaction is found absent in HCgp39 as the residue corresponding to Asp292 is replaced by Gly [8,44]. In HCgp39, Arg203 forms a hydrogen bond with Gln293, while such an interaction was absent in OVGP1 due to replacement of Arg203 by Ser210 residue. In SPC-40, both Arg203 and Gln293 are conserved, but no hydrogen bond formation occurs due to the unfavorable orientations of these residues. Hence, inspite of a closely related sequence similarity, various amino acid substitutions and conformational differences were observed among OVGP1 and its homologs displaying striking structural and functional differences.

### Evolutionary relationship of buffalo OVGP1 with other GH18 family members

**Figure 7:**
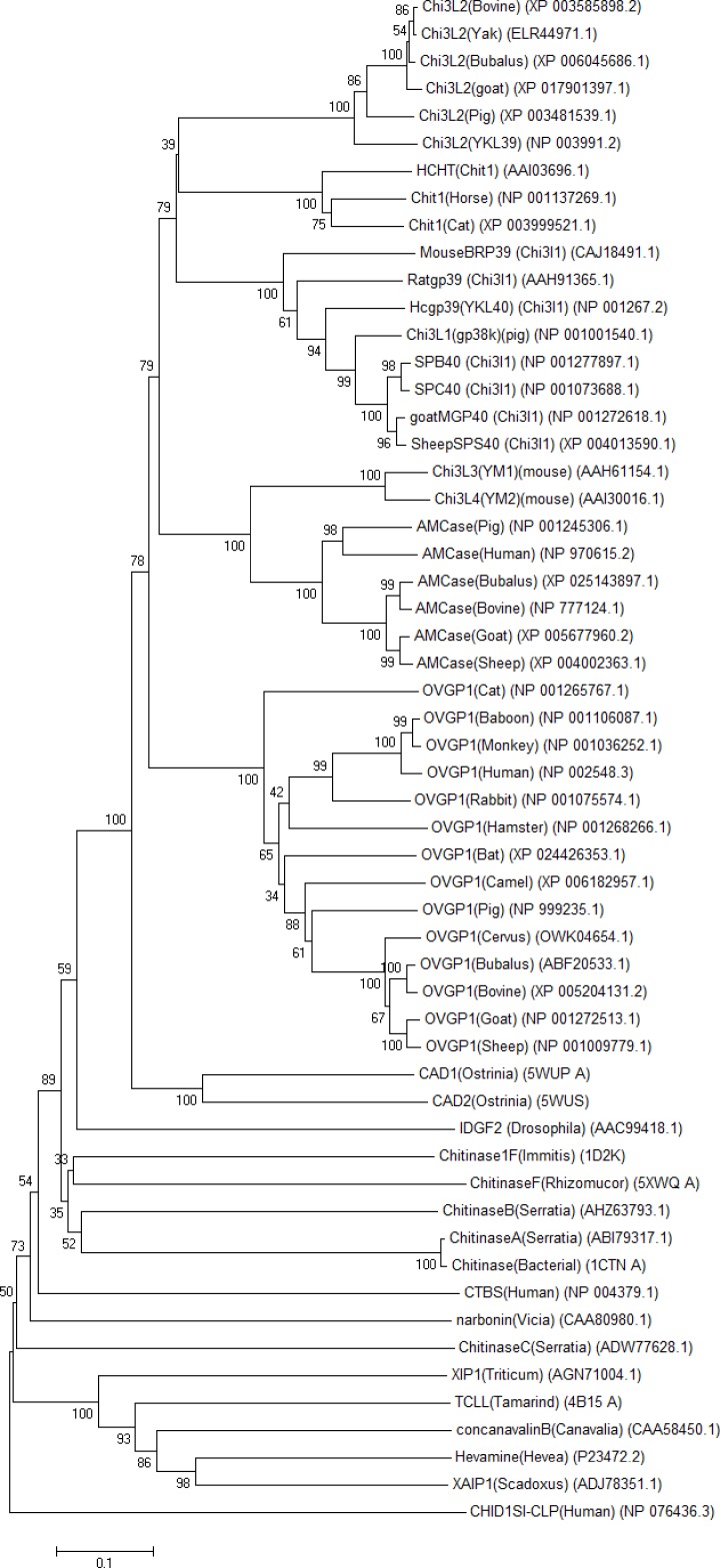
Evolutionary relationship ofOVGP1 in various species with other GH18 family members The tree was created from the deduced amino acid sequences by Neighbor Joining method in the MEGA 4.0 program. The tree was drawn to scale, and the numbers on the branches represent the confidence levels obtained from the bootstrap analysis (1000 replicates).

The evolutionary relationship of buffalo OVGP1 was examined by comparison with that of other species and various GH18 family members including both chitinases and chitinase-like proteins from mammals, plants, insects, bacteria and fungi. For a reliable phylogenetic tree construction, several amino acid substitution models were applied where *p*-distance model showed reliable bootstrap values for most of the important nodes. The tree suggests several gene duplication events in GH18 family leading to the segregation of various groups of paralogs. The duplication and divergence events led to the appearance of multiple forms of active chitinases and inactive chitinase-like proteins in various animals, human, bacterial, fungal and plant species (Figure 7). The duplication events suggest that OVGP1 possesses the closest evolutionary relationship with acidic mammalian chitinases (AMCases) and insect chitinases that is supported by higher bootstrap values. OVGP1 was observed to be most distant to plant CLPs and chitinases. The tree shows that OVGP1 in various species have been grouped into a separate clade. The acidic mammalian chitinases in human and animal species are also grouped into a single clade to which YM1 (Chi3L3) and YM2 (Chi3L4) form a sister clade. The inactive Chi3L1 and Chi3L2 class of CLPs; and active mammalian CHIT1 proteins form their separate individual groups.

## Discussion

OVGP1 belongs to the class of chitinase-like proteins of GH18 family due to their closeness in sequence homology except for the C-terminal region corresponding to mucin-like domain. The C-terminal domain might have emerged during evolution to decipher some specific functions to OVGP1 like protection from degradative proteases during reproductive processes. To date, no experimental data are available for OVGP1 in any of the species; therefore, we decided to determine the 3D structure of buffalo OVGP1 by molecular modeling and simulation studies.

Structural superpositions revealed that the chitinase-like domain of buffalo OVGP1 possesses a well-defined sugar binding groove as found in other members of GH18 family except for some amino acid substitutions. The groove architecture represented a similar pattern in central subsites as compared with the distal subsites where most of the substituted amino acids are present. Short chain saccharides occupied their positions in central subsites i.e. closer to the scissile glycosidic bond as compared with the longer chain oligosaccharides that interact with residues of both central and distal subsites.

OVGP1 binds to gametes inside the fallopian tube and influences their fertilization potential and subsequent development [29,45,46]. It has been suggested that the N-terminal chitinase-like domain of OVGP1 interacts with oligosaccharide moieties of oocyte zona pellucida whereas the C-terminal mucin-like domain that is densely glycosylated acts as a protective shield around the oocyte and early embryo against attacks from their environment [5]. Therefore, in an attempt to establish a structure–function relationship of buffalo OVGP1, we performed computational molecular docking and fluorescence quenching experiments using the sugar compounds that are generally found attached to the zona pellucida glycoproteins of oocyte. As revealed by both computational and experimental analysis, OVGP1 possesses ability to bind various saccharides/oligosaccharides. Inspite of the presence of various amino acid substitutions in the carbohydrate-binding groove, OVGP1 binds to different sugars by various hydrogen bonding and non-bonded contacts. This indicates that the replacement of residues in the binding groove of OVGP1 with residues of significantly different chemical nature does not influence the binding of substrates or they might have evolved for a different subset of ligands or receptors. Further, the OVGP1–(GlcNAc)_6_ complex displayed more number of strong hydrogen bonds as compared with other complexes. This excess of hydrogen bonding contributes to stabilize the longer chain OVGP1–(GlcNAc)_6_ complex as hydrogen bonds act as important contributors to protein stabilization as well as stabilization of protein–carbohydrate complexes [47]. Similar observations have been made for complex structures of some other GH18 family members with sugar ligands of different lengths [48]. During quenching experiments, a decrease in the fluorescence intensity was observed after incubation with different ligands that further supports the significant sugar-binding characteristics of OVGP1.

The TIM barrel fold of family 18 chitinases possesses a conserved catalytic DxDxE motif (Asp136, Asp138 and Glu140 in HCHT) [6] and a hydrolytic reaction is catalyzed by substrate-assisted mechanism [49]. The corresponding residues observed in OVGP1 are Asp116, Phe118 and Leu120, respectively. Therefore, the substitution of polar residues to non-polar residues strongly alters the overall conformation and physicochemical properties of the catalytic center that could lead to the loss of chitinase activity in OVGP1. However, OVGP1 is able to bind to the short chain as well as long chain saccharides/oligosaccharides in its well conserved sugar binding groove as revealed by our molecular docking and experimental binding studies. The key catalytic residue i.e. Glu140 in the catalytic triad of chitinases that has been replaced by Leu120 in OVGP1 acts as a proton donor to the scissile glycosidic bond in -1 to +1 subsite. The neighboring Asp138 residue in chitinases stabilizes the oxazolinium ion intermediate by orienting the N-acetyl group of the sugar for nucleophilic attack on the anomeric carbon. But this catalytic Asp138 residue has been replaced by a bulky and non-polar Phe118 residue in OVGP1. Although some other chitolectins like narbonin, XIP-1 [50,51] possess this key catalytic glutamate residue, but still they do not show the catalytic activity due to their involvement in forming salt bridges and polar contacts with other amino acids. The presence of two non-conservative substitutions in active site may lead to the inability of OVGP1 to hydrolyze sugars; however, it may use some of the structural elements of chitinases to mediate its own functions such as specific binding to different carbohydrate moieties. The amino acid substitutions in the catalytic center indicate that carbohydrates bound to OVGP1 are not hydrolyzed; however, it is able to act as a lectin. Some other members belonging to the class of chitinase-like proteins of family 18 show binding to saccharides/polysaccharides in a similar manner as observed in chitinases but possess variable mutations of three critical residues in the catalytic site and lack the ability to hydrolyze sugars [7,41,52–54]. Other possible reasons that may be attributed to the loss of catalytic activity of OVGP1 and other CLPs may include a difference in the subsite preference, inaccessibility of key residues to the scissile glycosidic bond, or some ligand induced conformational changes of various residues inside the sugar-binding groove. This needs further investigation in order to have strong evidences that can be inferred well from the crystal structures of mutant CLPs in complex with saccharides/oligosaccharides.

The disulfide bonds in buffalo OVGP1 formed by four cysteine residues are found highly conserved across various species except cattle, where five Cys residues have been reported [55]. The presence of two disulfide bridges have also been reported for other mammalian chitinase-like proteins such as MGP-40, SPS-40 and BRP-39 [7,9,42]. These disulfide bridges help in maintaining the integrity and thus, stabilization of the protein structure. Furthermore, these two disulfide bonds are located at similar positions as found in other homologs suggesting their importance in conserved functions. The conserved nature of two disulfide bonds between four Cys residues across the family 18 proteins provides an evidence of their evolutionary relationship.

The evolution of OVGP1 might have occurred in mammals sequentially. This evolutionary pathway most likely involved gene duplication events with mutations at active site leading to the emergence of OVGP1 chi-lectin. This could be evident from the sequence comparison of OVGP1 with active chitinases and similar evidences of evolutionary mechanisms have been reported in mammals, plants and insects [56]. There are various structural evidences that show that these chi-lectins adopt similar (β/α)_8_ barrel fold as found in GH18 chitinases but lack the chitinase activity due to active site mutations [7–9]. However, they are able to bind chitin and other carbohydrates with different specificities [41,42]. For instance, HCgp-39 (YKL-40) is able to bind chitin fragments without hydrolyzing it and Ym1 (Chi3L3) chi-lectin shows specificity toward N-acetyl glucosamine and heparin/heparan sulfate [57]. The physiological significance of these findings is not yet clear. Therefore, the gene duplication events followed by mutations at catalytic center of chitinases of GH18 family led to the emergence of a broad category of chitinase-like proteins [55]. These inactive chitinases have evolved with conserved structural fold with loss of their chitinolytic activity; however, they have acquired precise biological functions through interactions with various ligands. Therefore, inspite of losing their chitinase activity, chi-lectins utilize the existing scaffold of GH18 family to conquer new biological functions. This is well exemplified by OVGP1 protein that has been demonstrated by various studies to play its role in various reproductive functions [1,29,30]. An extended C-terminal repeat region characteristic of mucins has evolved in OVGP1; however, the existence of conserved structural topology of N-terminal domain is responsible for specific recognition of carbohydrates attached to glycoproteins of oocyte zona pellucida and sperm surface to perform precise reproductive functions. In view of other chito-lectins, the proteins encoded by the CHI3L1 and CHI3L2 genes are involved in tissue remodeling during inflammation and/or development [58,59]. The inactive chitinase i.e. XIP-I has been reported to inhibit fungal xylanases of GH10 and GH11 family [51]. Another chito-lectin i.e. XAIP1 acts as inhibitor of xylanases of family GH11 and α-amylases of family GH13 [52]. As revealed by the phylogenetic analysis in our study, OVGP1 has a close evolutionary relationship with IDGF2 (imaginal disc growth factor-2) from *Drosophila melanogaster* after insect chitinases. IDGF2 is a glycosylated inactive chitinase having a partially blocked cavity for binding to oligosaccharides; however, it has been proposed to be involved in cell proliferation functions [54]. The CHID1 gene that was observed to be distantly related to OVGP1 in our study encodes a stabilin1-interacting lysosomal chito-lectin (SI-CLP) of GH18 family [60]. Therefore, inspite of possessing a conserved structural motif, the CLPs of GH18 family have evolved to perform diverse biological functions.

## Conclusions

Characterization of 3D structure of OVGP1 revealed similarity to both chitinases and chitinase-like proteins of GH18 family. Both computational and experimental analysis reveals that OVGP1 is able to bind sugars of different lengths as observed in other family members. Sugar binding occurs via several hydrogen-bonding interactions of sugar-hydroxyl groups and hydrophobic interactions with the side chains of aromatic amino acid residues. The saccharides/oligosaccharides fit strikingly well in the binding groove of buffalo OVGP1. The mutation of active site residues from Asp to Phe118, Glu to Leu120 and other specific structural variations due to amino acid substitutions in the sugar-binding groove indicates a loss of chitinase activity as well as variation in the sugar binding affinity of buffalo OVGP1 in comparison with the chitin-hydrolyzing chitinases. Therefore, OVGP1 seems to be a purely binding protein that binds initially to the glycans and subsequently aid in protein–protein interactions. This indicates that the sequence and structural homologies of OVGP1 are retained during evolution but with dramatic alterations in its functions. The results of the present study are expected to enhance the understanding of the role of OVGP1 protein in various reproductive processes by binding to gametes.

## Availability of data and materials

The datasets during the present study are available from the corresponding authors on reasonable request.

## Supplementary Material

Supplementary Figures S1-S4 and Tables S1-S2

## Abbreviations

GH18: glycosyl hydrolase 18
MD: molecular dynamics
OF: oviductal fluid
OVGP1: oviduct-specific glycoprotein
