# Supplementary material for: Structural and functional characterization of buffalo oviduct-specific glycoprotein (OVGP1) expressed during estrous cycle
Source: Biosci Rep. 2019 Dec 10;39(12):BSR20191501. doi: 10.1042/BSR20191501 (PMC6904773; doi:10.1042/BSR20191501)
Supplement: Supplementary Figures S1-S4 and Tables S1-S2 [file BSR-2019-1501_supp.pdf]

Table S1: List of 10 possible templates selected for model building process (Out of a total of 104 hits identified by PSI-BLAST)

The 'Total score' in the second column is simply the product of the BLAST alignment score, the WHAT_CHECK quality score in the PDBFinder2 database and the target coverage. This makes sure that good template structures are used even if the alignment score is lower.

Template	Total score	BLAST E-value	Align score	Cover	ID	Resolution	Header
1	361.56	4e-125	840.0	69%	5WSI-A	1.49 A	XRAY Chitinase-3-like protein 1 [Bubalus bubalis] <CH3L1_BUBBU(22-383)> (361 residues with resolution 1.490), released 2016-12-21
2	352.01	2e-131	944.0	71%	3RME-A	1.80 A	XRAY Acidic mammalian chitinase [Homo sapiens] <CHIA_HUMAN(22-408)> (377 residues with quality score 0.523), released 2011-08-24
3	339.00	8e-144	950.0	69%	4WKA-A	0.95 A	XRAY Chitotriosidase-1 [Homo sapiens] <CHIT1_HUMAN(22-386)> (369 residues with quality score 0.514), released 2015-07-08
4	334.46	7e-137	967.0	69%	1HKK-A	1.85 A	XRAY Chitotriosidase-1 [Homo sapiens] <CHIT1_HUMAN(22-385)> (364 residues with quality score 0.500), released 2004-03-11
5	318.82	7e-131	946.0	71%	2YBU-B	2.25 A	XRAY Acidic mammalian chitinase [Homo sapiens] <CHIA_HUMAN(21-398)> (376 residues with quality score 0.474), released 2011-06-08
6	304.29	8e-119	864.0	69%	4P8V-A	1.64 A	XRAY Chitinase-3-like protein 2 [Homo sapiens] <CH3L2_HUMAN(27-390)> (365 residues with quality score 0.512), released 2014-12-03
7	303.09	5e-119	858.0	69%	4AY1-A	1.95 A	XRAY Chitinase-3-like protein 2 [Homo sapiens] <CH3L2_HUMAN(26-390)> (364 residues with quality score 0.515), released 2012-08-08
8	298.26	2e-117	819.0	71%	1VF8-A	1.31 A	XRAY Chitinase-like protein 3 [Mus musculus] <CHIL3_MOUSE(22-398)> (373 residues with quality score 0.515), released 2005-03-15
9	296.88	4e-124	847.0	69%	2PI6-A	1.65 A	XRAY Chitinase-3-like protein 1 [Ovis aries] <CH3L1_SHEEP(1-360)> (361 residues with quality score 0.511), released 2007-05-01
10	115.69	3e-084	267.0	95%	4TXG-A	1.75 A	XRAY Probable chitinase [Chromobacterium violaceum] <Q7NSV3_CHRVO(1-791)> (679 residues with quality score 0.458), released 2015-10-14

Table S2: List of models (generated from alignment variants of different templates) used to build to the final hybrid model. YASARA tried to combine the best parts of the 43 models to obtain a hybrid model, hoping to increase the accuracy beyond each of the contributors. The following fragments were copied from other models (The first transfer is simply the initial model considered most suitable for hybridization). Different alignment variants are labeled as A01, A02 ….etc.

Transfer	First residue	Last residue	Length	From model	Score
1	1	361	361	1HKK-A01	**-3.247**
2	359	514	156	4TXG-A04	**-1.712** accepted
3	358	369	12	4TXG-A02	**-1.696** accepted
4	358	369	12	4TXG-A02	**-1.687** accepted
5	187	219	33	3RME-A03	**-1.643** accepted
6	358	369	12	4TXG-A02	**-1.616** accepted
7	358	369	12	4TXG-A02	**-1.606** accepted
8	358	369	12	4TXG-A02	-1.620 rejected
9	360	370	11	4TXG-A05	-1.608 rejected
10	45	55	11	2PI6-A02	-1.610 rejected
11	137	151	15	1VF8-A01	-1.613 rejected
12	279	286	8	1VF8-A02	-1.615 rejected
13	282	287	6	2YBU-B02	-1.625 rejected
14	443	457	15	4TXG-A02	-1.610 rejected
15	168	173	6	2PI6-A03	-1.606 rejected
16	169	174	6	2PI6-A03	-1.621 rejected
17	147	155	9	4TXG-A05	-1.618 rejected

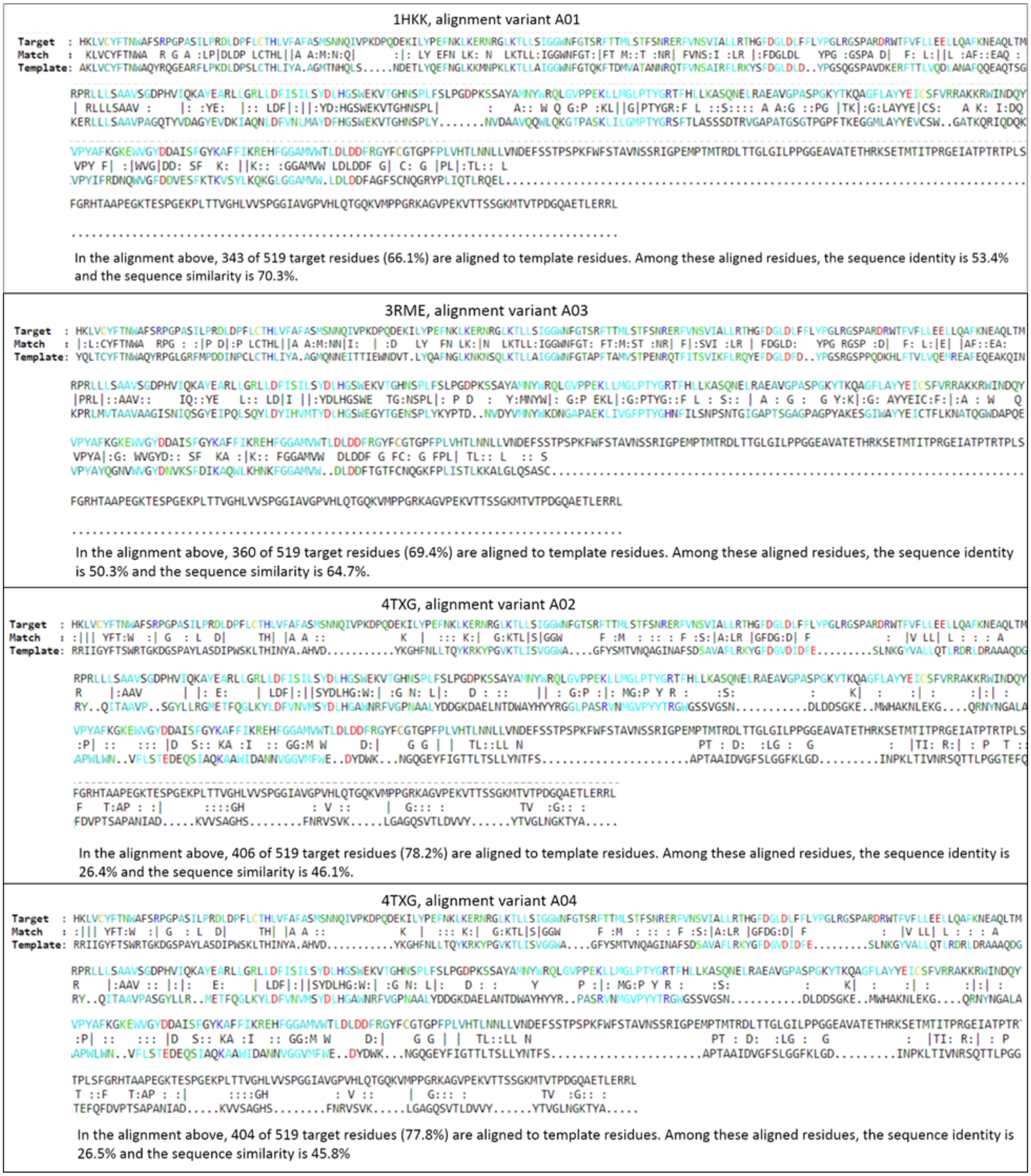

Fig. S1: Sequence alignments used to generate the final hybrid model of full-length OVGP1 from three templates i.e. 1HKK, 3RME and 4TXG.

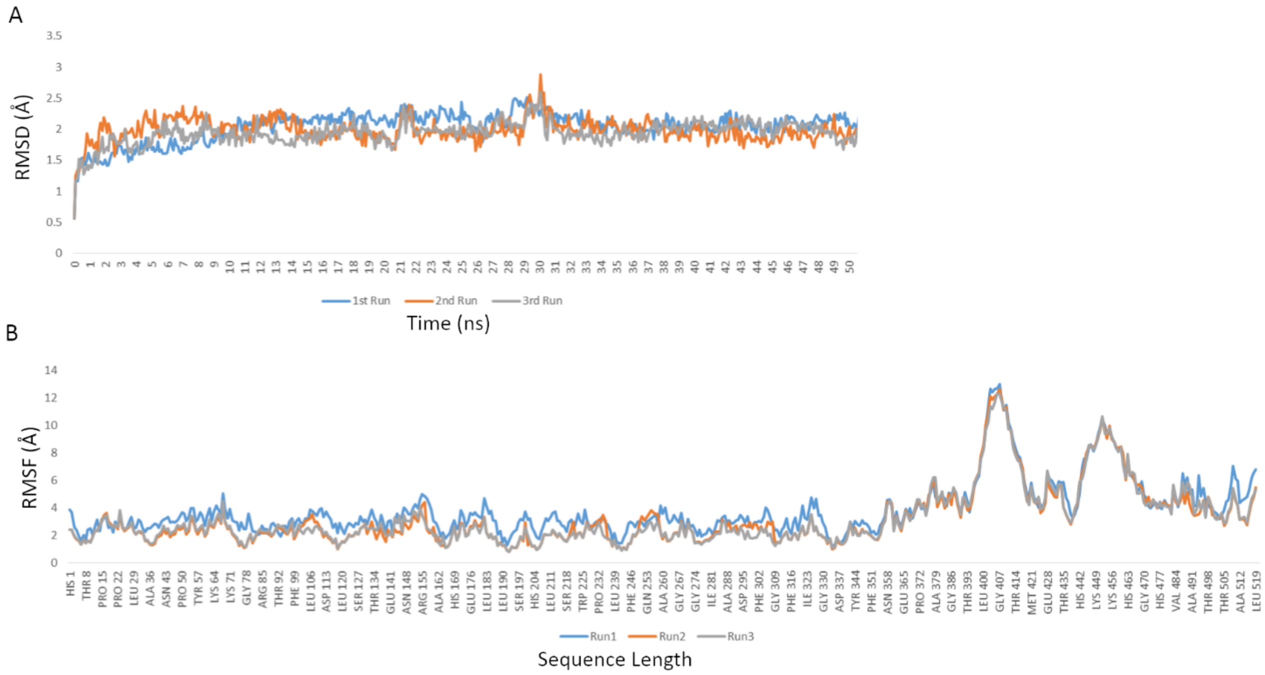

Fig. S2: RMSD and RMSF calculations for buffalo OVGP1 model structure. (A) Cα RMSD during 50 ns atomistic simulation showing the structural stability and simulation integrity during three repeated runs from the same starting structure. **(B)** Observed RMSF per residue of OVGP1 over the 50 ns trajectory during three repeated runs, which indicates the structural rigidity and measures the flexibility of the polypeptide chain. Region between 362-592 residues representing the C- terminal mucin-like domain shows a considerable flexibility.

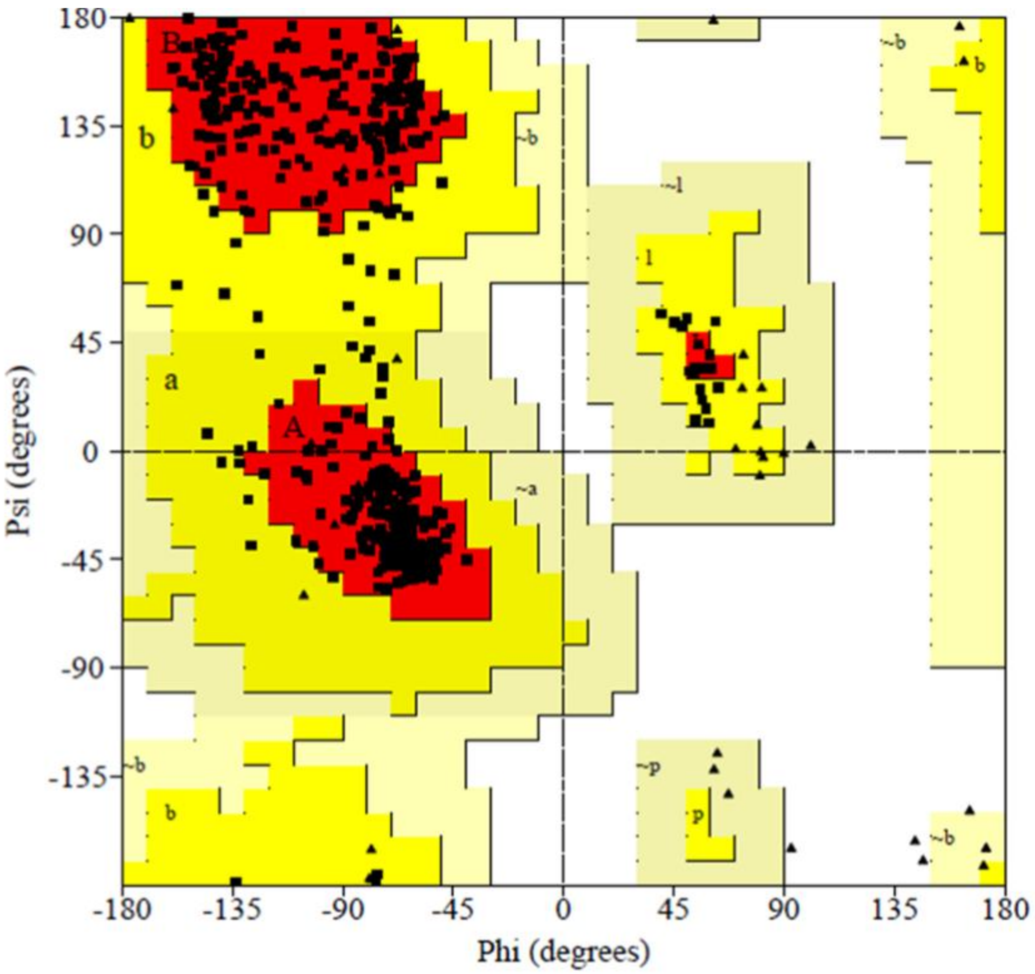

Fig. S3: Ramachandran plot of the φ–ψ distribution of modelled OVGP1. The red, brown and yellow regions represent the most favored, additionally allowed and generously allowed regions as defined by PROCHECK.

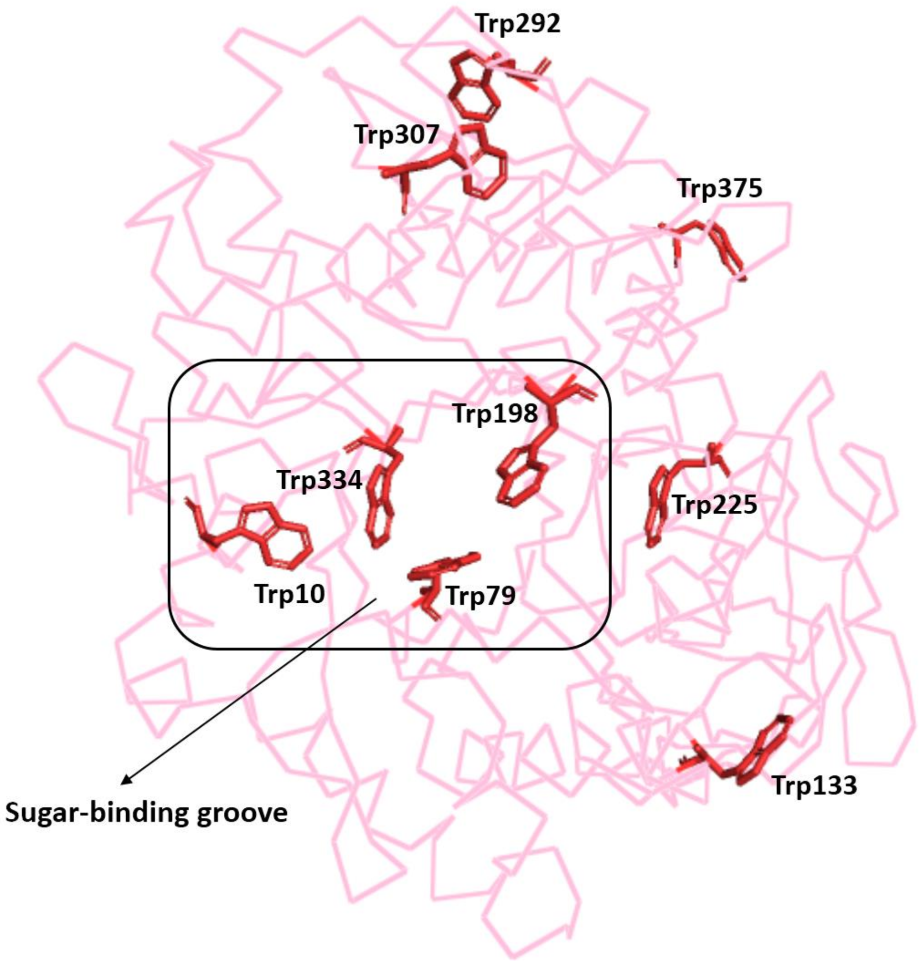

Fig. S4: Overall display of buffalo OVGP1 showing the positions of all native tryptophan residues. The residues Trp10, Trp79, Trp198 and Trp334 within the black squared box are located within the sugar-binding groove and involved in sugar-binding. The remaining five residues (outside the box) are located away from the binding site.
